# Intake of a Mixture of Sake Cake and Rice Malt Increases Mucin Levels and Changes in Intestinal Microbiota in Mice

**DOI:** 10.3390/nu12020449

**Published:** 2020-02-11

**Authors:** Shinpei Kawakami, Ryouichi Ito, Hiroko Maruki-Uchida, Asuka Kamei, Akihito Yasuoka, Tsudoi Toyoda, Tomoko Ishijima, Eisaku Nishimura, Minoru Morita, Masahiko Sai, Keiko Abe, Shinji Okada

**Affiliations:** 1 Health Science Research Center, Morinaga & Co., Ltd., 2-1-1 Shimosueyoshi, Tsurumi-ku, Yokohama 230-8504, Japan; r-ito-ih@morinaga.co.jp (R.I.); h-uchida-ji@morinaga.co.jp (H.M.-U.); e-nishimura-ie@morinaga.co.jp (E.N.); m-morita-je@morinaga.co.jp (M.M.); m-sai-ia@morinaga.co.jp (M.S.); 2 Kanagawa Institute of Industrial Science and Technology, LiSE 4F C-4 3-25-13 Tonomachi, Kawasaki-ku, Kawasaki, Kanagawa 210-0821, Japan; fp-kamei@newkast.or.jp (A.K.); ayasuoka@mail.ecc.u-tokyo.ac.jp (A.Y.); aka7308@mail.ecc.u-tokyo.ac.jp (K.A.); 3 Graduate School of Agricultural and Life Sciences, The University of Tokyo, 1-1-1 Yayoi, Bunkyo-ku, Tokyo 113-8657, Japan; toyoda-t@takasaki-u.ac.jp (T.T.); aishiji@mail.ecc.u-tokyo.ac.jp (T.I.); asoka@mail.ecc.u-tokyo.ac.jp (S.O.)

**Keywords:** sake cake, rice malt, mucin, microbiota

## Abstract

Amazake is a traditional Japanese beverage. Its main ingredients are sake cake and rice malt. In this study, we examined the effect of sake cake and rice malt on the intestinal barrier function and gut microbiota. BALB/c mice were fed a control diet or a diet containing a mixture of sake cake and rice malt powder (SRP) for four weeks. Fecal IgA values did not change between groups, but the fecal mucin level was significantly greater in the SRP-fed group. Gene expression analysis in the ileum by real-time PCR demonstrated *Muc2* expression did not change, while the *Muc3* expression was upregulated in the SRP-fed group. Furthermore, microbiota analysis demonstrated a change by SRP intake at the family level, and the proportion of *Lactobacillaceae* significantly increased in the SRP-fed group. At the genus level, the proportion of *Lactobacillus* also significantly increased in the SRP-fed group. These results suggest that the intake of a mixture of sake cake and rice malt improves intestinal barrier function by increasing mucin levels and inducing changes in intestinal microbiota.

## 1. Introduction

The functions of the small intestine include the digestion of food and absorption of nutrients. The intestine is also exposed to pathogenic bacteria and their toxins. Thus, the intestine has a robust immune system to protect the body against these pathogenic factors from the outside [1]. The intestinal tract is covered with a mucosal layer containing mucin as a main component. Mucin and immunoglobulin A (IgA) are important components responsible for intestinal barrier function, which prevent bacterial invasion and toxins [2,3]. The gut microbiota is also an important gut environment factor, and the effects of dietary nutrition on the intestinal barriers’ function and gut microbiota have been studied. Dietary fiber and some polyphenolic components have been reported to increase the concentrations of intestinal IgA and mucin and to improve the gut barrier function [4,5] with yogurt, one of the famous fermented foods, influencing the intestinal microbiota [6].

Amazake is a sweet traditional Japanese beverage originating from “Amanotamuzake,” mentioned in Nihonshoki (Chronicles of Japan). Amazake is classified according to its production method. “Rice malt amazake” is made by saccharification using rice malt, and “sake cake amazake” is made from sake cake. In addition, amazake made from both rice malt and sake cake is widely consumed in Japan. Sake cake is a byproduct of sake brewing and traditionally used for soup (kasu-jiru) and pickled fish (kasu-zuke). Rice malt is made by fermenting steamed rice with fungus and is widely used in traditional Japanese foods such as miso and soy sauce. Amazake contains many nutrients, while rice malt amazake contains over 300 compounds, including sugars, amino acids, organic acids, and vitamin B complex [7]. To combat malnutrition, which is a frequent complication in patients with chronic liver disease, a clinical trial of amazake made from rice malt intake was conducted. The trial, which administered amazake as a late evening snack by liver cirrhosis patients, showed amazake was effective at improving the quality of life (QOL) of those patients [8].

Sake cake contains digestion-resistant protein [9], and its intake was reported to increase fecal IgA and mucins for a desirable microbiota in rats fed with a high-fat diet [10]. Sake cake contains a residue of sake yeast, which provides numerous health benefits. As previously reported, the administration of dietary yeast increases the population of *Lactobacillales* and decreases the *Bacteroides* in mice [11]. Another report demonstrated that the intake of yeast changes the gut microbial composition, and the genus *Lactobacillus* is only detected in yeast-fed groups [12] Also, the intake of glycosylceramide, derived from rice malt, was reported to increase *Blautia coccoides* in mice [13]. From these results, amazake made of both rice malt and sake cake was expected to improve the intestinal function. However, the potential health benefits of these factors toward an intestinal environment have not been clarified. In this study, we investigated the effects of intake of a mixture of sake cake and rice malt as main ingredients of amazake on the intestinal barrier function as well as the intestinal microbiota.

## 2. Materials and Methods

### 2.1. Preparation of a Mixture of Sake Cake and Rice Malt

Sake cake and rice malt were obtained from Morinaga & Co., Ltd. (Tokyo, Japan). One part of rice malt was dissolved in two parts of hot water and incubated for 1 h at 60 °C. Two parts of sake cake were added to the rice malt solution and mixed, then the mixture was freeze-dried. The freeze-dried sample was powdered to obtain the sake cake and rice malt powder (SRP). The nutritional components of this powder were analyzed and are shown in Table 1. The total dietary fiber was analyzed using the enzymatic-gravimetric method (Association of Official Analytical Chemists (AOAC) 985.29 method), being the sum of high molecular weight water-soluble dietary fiber and insoluble dietary fiber.

### 2.2. Animals and Treatments

All animal experiments were conducted with strict compliance with the Guidelines for Proper Conduct of Animal Experiments by the Science Council of Japan. The experimental protocols and procedures were approved by the Animal Experimental Committee of Morinaga & Co., Ltd. (permission number: 168-01-004). This experiment was approved and conducted in 2015.

Eight-week-old male BALB/c mice (Japan SLC, Shizuoka, Japan) were housed individually in plastic cages in a room with controlled temperature (21–25 °C) and humidity (40%–60%). Fourteen mice were maintained on a 12-h dark/light cycle with artificial lighting. After one week of acclimatization, the mice were divided into two groups with similar average body weights (n = 7). Thereafter, the mice were fed the experimental diets with free access to drinking water. The control group was fed a normal diet (AIN-93G; CLEA JAPAN Inc., Tokyo, Japan), while the SRP group was fed a diet containing 10% SRP instead of 10% carbohydrate. The body weight and food intake of mice were measured twice per week. The diet composition is shown in Table 2.

During feeding, 24-h total feces were collected at the start of SRP ingestion (week 0), and collected at the end of each subsequent week, until week four. Feces were collected from the bottom of the cage and stored at −20 °C until analysis. After four weeks of feeding, the mice were fasted overnight, then all mice were sacrificed under anesthesia. A 4-cm-length section of the lower ileum was dissected, washed with saline, and everted. Epithelial tissue were collected, then rapidly frozen with liquid nitrogen. Epithelial tissue samples were stored at −80 °C until RNA extraction. The epididymal and perirenal fats, liver, kidney, spleen, and heart were extracted and weighed.

### 2.3. Analysis of Fecal IgA and Mucin

The quantifications of fecal IgA and mucin were performed by Cosmo Bio Co., Ltd. (Tokyo, Japan). Fecal IgA contents were quantified by enzyme-linked immunosorbent assay (ELISA) using a mouse IgA ELISA Quantitation Set (Bethyl Laboratories, Inc., Montgomery, TX, USA). Fecal mucin contents were quantified using a Fecal Mucin assay kit (Cosmo Bio Co., Ltd.) that discriminates O-linked glycoproteins (mucins) from N-linked glycoproteins [14]. Approximately 100 mg and 50 mg of feces were used for mucin and IgA analysis, respectively, and the values were expressed as values per feces weight.

### 2.4. RNA Extraction and qRT-PCR

Total RNA samples were individually extracted from the ileums of all mice using the TRIzol reagent (Thermo Fisher Scientific, Waltham, MA, USA) and purified with the RNeasy Mini kit and RNase-free DNase set (Qiagen, Hilden, Germany) according to the manufacturer’s instructions. Quantifications of mRNA expression were performed as previously described [15]. Purified total RNA samples were reverse transcribed into complementary DNA by a High-Capacity cDNA Reverse Transcription Kit (Thermo Fisher Scientific), and real-time polymerase chain reactions (PCR) were performed using Light Cycler 480 Real-Time PCR (Roche Diagnostics, Mannheim, Germany) by Light Cycler 480 Probes Master and Universal Probe Library probes. PCR primers used were forward (5′-AAGTGAAGACCGAGATTGTGC-3′) and reverse (5′-GTGCACACACACACCCTTG-3′) for *Muc2*, forward (5′-TTCTATGGGCCACGGTGT-3′) and reverse (5′-ACTGGTTACTGTCACACTCACTCC-3′) for *Muc3*, and forward (5′-AGCCACATCGCTCAGACAC-3′) and reverse (5′-GCCCAATACGACCAAATCC-3′) for glyceraldehyde 3-phosphate dehydrogenase (*Gapdh*). Target gene expression was normalized to *Gapdh* mRNA expression levels.

### 2.5. Analysis of Microbiota

Fecal microbiota analysis was performed by TechnoSuruga Laboratory Co., Ltd. (Shizuoka, Japan). Extraction of DNA from fecal samples and 16S rRNA sequencing using the MiSeq system (Illumina, San Diego, CA, USA) was performed according to a previously described method [16]. The V3–V4 regions of 16S rRNA were PCR amplified from microbial genomic DNA using the Prokaryotes universal primers (Pro341F/Pro805R) [16] and the dual-index method [17]. Barcoded amplicons were sequenced using the paired-end, 2 × 284-bp cycle run on the MiSeq system with the MiSeq Reagent Kit version 3 (600 Cycle) chemistry.

Bioinformatics analysis was performed according to a previously described method [16]. The overlapping paired-end reads were merged using the fastq-join program with default settings [18]. The reads were processed with quality and chimera filtering as follows. Only reads that had quality value (QV) scores of 20 for over 99% of the sequence were extracted and chimeric sequences were removed using the usearch6.1 [19]. Nonchimeric reads were submitted for 16S rDNA-based taxonomic analysis using the Ribosomal Database Project (RDP) Multiclassifier tool [20].

### 2.6. Statistical Analysis

All data are presented as mean ± standard error of the mean (SEM). The difference between two groups was analyzed using the Student’s *t*-test or Welch’s *t*-test according to the results of the *F* test. *p* < 0.05 was considered statistically significant. These statistical analyzes were performed using SPSS software version 22 (SPSS, Tokyo, Japan).

## 3. Results

### 3.1. Body Weight, Food Intake, and Tissue Weight

To examine the effects of intake of a mixture of sake cake and rice malt on intestinal functions, mice were fed a control diet or a diet containing SRP. After four weeks of feeding, no significant difference in their body weight, food intake, and their tissue weight between the control and SRP group were seen (Table 3).

### 3.2. Analysis of Fecal IgA and Mucin

**Figure 1:**
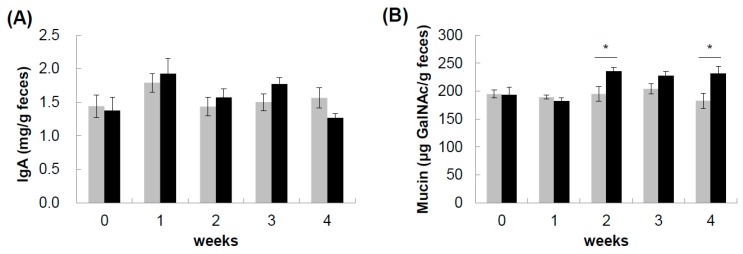
Fecal IgA and mucin contents. Feces from mice fed a control diet (control, gray bar) or a diet with sake cake and rice malt (SRP, black bar) were collected every week during four weeks of feeding. (**A**) IgA contents and (**B**) mucin contents in feces were measured. Data are shown as the mean ± SEM (*n* = 7). * *p* < 0.05.

To investigate the effect of sake cake and rice malt on gut barrier function, IgA and mucin contents in feces were collected for up to four weeks and analyzed. There was no difference in the level of fecal IgA between groups (Figure 1A). In contrast, the fecal mucin value was significantly larger in SRP group than in the control group after two weeks and four weeks of feeding (Figure 1B).

### 3.3. qRT-PCR Analysis of Mucin-Related Gene Expression

**Figure 2:**
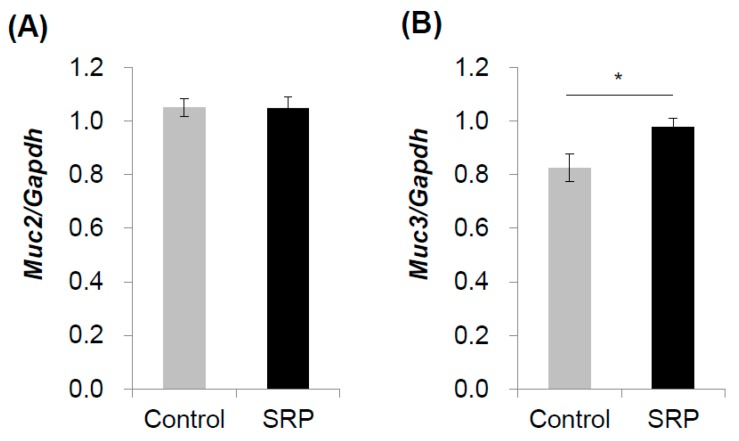
Quantification of *Muc2* and *Muc3* gene expression in the lower ileum. Relative gene expression of (**A**) *Muc2* and (**B**) *Muc3* were investigated in the lower ileum of mice fed a control diet (control) and a diet with sake cake and rice malt (SRP) for four weeks by a qRT-PCR method. The expression ratios of target genes to *Gapdh* were calculated. Data are shown as the mean ± SEM (*n* = 7). * *p* < 0.05.

The increased mucin content in the feces of the SRP mice group suggests that intake of sake cake and rice malt enhanced mucin-related gene expression in the gut. Thus, the expressions of two major intestinal mucin genes, *Muc2* and *Muc3*, were quantified by qRT-PCR analysis in the ileum. The *Muc2* mRNA expression did not change between groups (Figure 2A), while the *Muc3* mRNA expression in SRP group increased significantly than in the control group (Figure 2B).

### 3.4. Analysis of Fecal Microbiota

**Figure 3:**
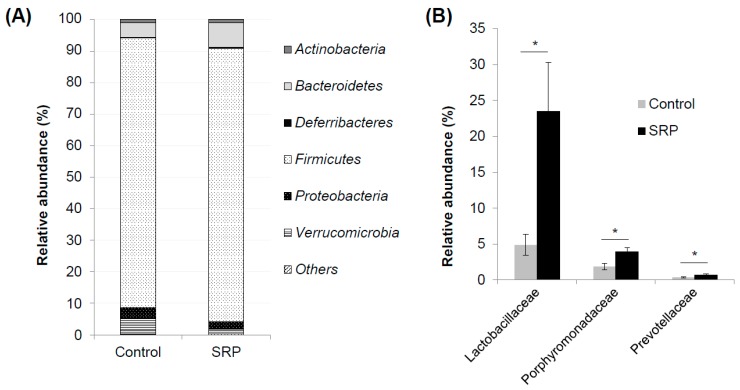
Gut microbiota analyzed by 16S rRNA gene sequencing using feces of mice fed a control diet (control) and a diet with sake cake and rice malt (SRP) for four weeks. (**A**) The composition of gut microbiota at the phylum level. (**B**) Significantly different taxa at the family level. Data are shown as the mean ± SEM (*n* = 7). * *p* < 0.05.

To investigate whether an intake of a mixture of sake cake and rice malt influenced intestinal microbiota, we compared the relative abundance of detected taxa in each group using next-generation sequencing microbiota analysis. The proportion of bacteria in feces collected after four weeks feeding in the control and SRP groups provided the results shown in Figure 3. At the phylum level, significant differences in the microbiota between the two groups were not observed (Figure 3A). At the family level, the proportion of *Lactobacillaceae*, *Porphyromonadaceae*, and *Prevotellaceae* were significantly larger in the SRP group than the control group (Figure 3B). The proportion of bacteria at the genus level is shown in Table 4. At the genus level, the proportion of *Alloprevotella, Barnesiella, Coprobacter* and *Lactobacillus* were significantly larger in the SRP group than the control group.

## 4. Discussion

Here, we investigated the effect of intake of a mixture of sake cake and rice malt (the main ingredients of amazake) on the gut barrier function. Our findings show that intake of an SRP diet for four weeks increased fecal mucin quantities. The mucus layer in the intestinal tract forms a physical barrier to protect the epithelial cells from potential luminal attacks from the likes of pathogenic bacteria or toxins. Mucin-deficient model mice showed mucosal thickening, enhanced colon inflammation, and enhanced susceptibility to colitis [21]. Enhancing mucin secretion can give protection against colitis, and previous reports, which have shown that dietary food improves mucin secretion [4,5,22], give evidence to this. It has been reported that changes in the properties of the mucin layer in the small intestine may affect the absorption of ions [23]. In our study, intake of SRP increased the fecal mucin. Therefore, sake cake and rice malt have the potential to improve the gut barrier function by enhancing mucin secretion.

Changes in mucin gene expression in the ileum were investigated, and the *Muc3* mRNA expression increased significantly with SRP intake. Muc3 is one of the prominent mucin genes expressed in the intestine [24]. *Muc3* is expressed in both goblet cells and enterocytes [25] and undergoes alternative splicing to encode a family of proteins that can be membrane bound or secreted [26]. Therefore, the upregulation of *Muc3* mRNA would be one factor contributing to the enhanced mucin secretion detected in the SRP mice groups’ feces.

Changes in the microbiota because of SRP intake were examined. Four weeks after SRP intake, the proportion of *Lactobacillaceae*, *Porphyromonadaceae*, and *Prevotellaceae* were significantly greater in the SRP group at the family level. In particular, the proportion of *Lactobacillaceae* was dramatically increased due to SRP intake. *Lactobacillaceae* are a family of lactic acid bacteria, which are considered beneficial in the intestinal environment. At the genus level, the populations of *Lactobacillus* were significantly larger in the SRP group than in the control group. These results suggest that sake cake and rice malt can act as prebiotics, as sake cake contains sake yeast residue. Likewise, the administration of *Candida kefyr*, one of the dietary yeasts, increases the population of *Lactobacillales* in mice [11]. Therefore, the residue of sake yeast in sake cake may have the potential to increase the population of *Lactobacillaceae*. The proportion of *Porphyromonadaceae* and *Prevotellaceae* also increased due to SRP intake. It has been reported that the *Barnesiella* genus belongs to the family of *Porphyromonadaceae* and confers resistance to indicated intestinal domination with vancomycin-resistant *Enterococcus* [27]. The proportion of *Barnesiella* also increased due to SRP intake, and this change in the *Porphyromonadaceae* and *Barnesiella* due to SRP intake might help prevent the transmission of resistant bacteria. *Alloprevotella* is a genus from the family *Prevotellaceae*, and *Alloprevotella* increased due to SRP intake. However, the function of *Alloprevotella* in the intestine is not well understood, and intake of dietary fiber has been reported to increase the ratio of *Alloprevotella* [28]. These changes in the microbiota due to SRP intake have a potential to contribute to the improvement of the intestinal barrier function and our whole-body health through the improvement of the intestinal environment.

Intestinal bacteria reside in the surface mucus layer [29]. The study of transgenic mice that promoted mucin production revealed that the mucin layer was more robust and housed a significantly greater abundance of *Lactobacillus spp.* in the ileum [30]. Therefore, the enhancement of mucin production due to SRP intake might increase the population of *Lactobacillaceae* in the intestine. On the other hand, the application of *Lactobacillus rhamnosus* GG culture supernatant has been reported to upregulate mucin production and protect from *E. coli* infection in rats [31]. This result shows another possibility that the intake of SRP increases the intestinal *Lactobacillaceae* population, which may influence the mucin secretion system and enhance mucin secretion in the intestine. Mucin-degrading bacteria, such as *Akkermansia muciniphila*, exist in the intestine [32]. Although the microflora analysis at the species level has not been carried out and cannot be concluded, significant differences in genus *Akkermansia* were not observed between Control and SRP groups. It remains unclear from this study what causes mucin increase and a change of microbiota by SRP intake. Still, mucin production and microbiota may interact with each other to constitute a better condition through SRP intake.

In this study, an increase in mucin quantity and the change in microbiota were observed due to SRP intake in mice. The intake of amazake containing both sake cake and rice malt might also improve intestinal barrier function by increasing mucin levels in humans, thus protecting our body from harmful bacteria and toxins. Thus, some components of sake cake and rice malt are expected to improve intestinal barrier functions and improve the intestinal environment. However, these components were not elucidated in this study. The generation of diverse metabolites of sugar, organic acid, phenolic acid, and amino acid was previously reported during the saccharification of rice malt [33]. Among such metabolites, there may be physiological functional substances that exhibit an improvement in intestinal barrier function. Dietary fiber has been reported to increase mucin secretion [4]. Although detailed components of fiber in SRP have not been clarified, SRP contains dietary fiber. Moreover, short-chain fatty acids, which are formed in the colon following the fermentation of dietary fibers, also increase mucin secretion [34]. Such potential mediators that affect mucin secretion may contribute to mucin enhancement by SRP ingestion. Further studies are required to clarify what constituents in sake cake and rice malt contribute to the gut barrier function and microbiota.

## Figures and Tables

**Table 1:** The nutritional component of powder containing both sake cake and rice malt.

	Content (g/100 g Dry Basis)
Carbohydrate	79.1
Protein	14.4
Fat	2.2
Dietary fiber	3.2
Alcohol	0.7
Others	0.4

**Table 2:** Composition of the experimental diet.

Ingredient (g/kg Diet)	Control	SRP
Sake cake and rice malt mixed powder	0.0	100.0
Corn starch	397.5	297.5
Casein	200.0	200.0
Maltodextrin	132.0	132.0
Sucrose	100.0	100.0
Soybean oil	70.0	70.0
Powdered cellulose	50.0	50.0
AIN-93G mineral mix	35.0	35.0
AIN-93 vitamin mix	10.0	10.0
L-cystine	3.0	3.0
Choline bitartrate	2.5	2.5
t-butylhydroquinone	0.014	0.014

**Table 3:** Body weight gain, food intake, and tissue weights.

	Control	SRP
Final body weight (g)	28.9 ± 0.6	29.1 ± 0.6
Body weight gain (g)	5.8 ± 0.5	6.0 ± 0.6
Food intake (g/day)	4.2 ± 0.1	4.2 ± 0.1
Epididymal and perirenal fat (g)	0.53 ± 0.06	0.48 ± 0.05
Liver (g)	1.12 ± 0.03	1.12 ± 0.03
Kidney (g)	0.46 ± 0.01	0.49 ± 0.02
Spleen (g)	0.09 ± 0.01	0.09 ± 0.00
Heart (g)	0.14 ± 0.00	0.14 ± 0.00

Data are shown as the mean ± SEM (*n* = 7).

**Table 4:** Genus level taxonomic distributions of the microbiota in the feces.

	Control	SRP	*p* Value
*Akkermansia*	5.08 ± 2.62	1.58 ± 0.91	0.230
*Alkalitalea*	0.36 ± 0.11	0.57 ± 0.14	0.256
*Allobaculum*	26.47 ± 4.19	20.82 ± 4.25	0.363
*Alloprevotella*	0.36 ± 0.08	0.70 ± 0.12	0.038
*Bacteroides*	1.75 ± 0.41	2.25 ± 0.54	0.479
*Barnesiella*	1.58 ± 0.37	3.03 ± 0.44	0.026
*Bilophila*	2.98 ± 1.13	1.97 ± 0.39	0.414
*Clostridium IV*	1.05 ± 0.17	0.68 ± 0.13	0.103
*Clostridium XlVa*	4.95 ± 0.79	5.05 ± 0.98	0.944
*Clostridium sensu stricto*	8.30 ± 5.44	2.22 ± 1.54	0.303
*Coprobacter*	0.17 ± 0.04	0.72 ± 0.14	0.003
*Coprococcus*	0.24 ± 0.11	0.26 ± 0.05	0.875
*Desulfovibrio*	0.24 ± 0.06	0.28 ± 0.05	0.560
*Dorea*	0.85 ± 0.15	0.88 ± 0.21	0.895
*Eisenbergiella*	0.23 ± 0.04	0.41 ± 0.11	0.162
*Enterorhabdus*	0.92 ± 0.06	0.88 ± 0.13	0.792
*Eubacterium*	1.90 ± 0.36	1.18 ± 0.22	0.113
*Flavonifractor*	0.90 ± 0.17	0.66 ± 0.29	0.475
*Lachnospiracea_incertae_sedis*	2.31 ± 0.64	2.27 ± 0.36	0.965
*Lactobacillus*	4.85 ± 1.45	23.42 ± 6.78	0.020
*Lactococcus*	27.55 ± 2.36	22.37 ± 3.34	0.230
*Lactonifactor*	0.28 ± 0.15	0.77 ± 0.30	0.167
*Marvinbryantia*	0.45 ± 0.13	1.14 ± 0.40	0.126
*Moryella*	1.16 ± 0.33	1.35 ± 0.43	0.726
*Mucispirillum*	0.13 ± 0.03	0.27 ± 0.12	0.254
*Oscillibacter*	0.32 ± 0.15	0.31 ± 0.09	0.961
*Pseudoflavonifractor*	0.41 ± 0.11	0.33 ± 0.11	0.593
*Ruminococcus2*	0.65 ± 0.07	0.39 ± 0.10	0.060
*Turicibacter*	0.82 ± 0.81	0.31 ± 0.31	0.573

Relative abundance (%) are shown as the mean ± SEM (*n* = 7). The genera that has an abundance higher than 0.2% are shown.
